# Editorial: Towards a better understanding of hemophagocytic lymphohistiocytosis

**DOI:** 10.3389/fimmu.2024.1385487

**Published:** 2024-04-09

**Authors:** Sabrin Albeituni

**Affiliations:** Department of Oncology, St. Jude Children’s Research Hospital, Memphis, TN, United States

**Keywords:** hemophagocytic lymphohistiocytosis, cytokines, primary HLH, Secondary HLH, Epstein-Barr virus (EBV), ruxolitinib

Hemophagocytic lymphohistiocytosis (HLH) is a fatal inflammatory syndrome characterized by excessive activation of immune cells, including myeloid and T cells that overproduce excessive levels of pro-inflammatory cytokines (1). HLH is classified into two types: primary (familial) and secondary (acquired) HLH. In the context of rheumatic diseases, especially in systemic juvenile idiopathic arthritis (SJIA), secondary HLH is also referred to as macrophage activation syndrome (MAS). Primary HLH is caused by mutations in genes essential for the cytolytic function of cytotoxic cells. Variants in genes important for NK and T cell cytotoxicity have been reported to cause primary HLH, such as, *PRF1, UNC13D, STXBP2, STX11, RAB27A, LYST, AP3B1, SH2D1a*, and *XIAP/BIRC4* (2). Secondary HLH occurs secondary to malignancy, autoimmune diseases, severe infections, and rheumatic diseases (1). Recent studies have been unraveling monogenic causes for secondary HLH, including an activating mutation in *NLRC4* (NLRC4-MAS) which results in induced levels of IL-18 (3). As elevated levels of IL-18 are elevated in MAS (4–6), inhibition of IL-18 with recombinant human IL-18 binding protein, was reported to alleviate symptoms in a patient with NLRC4-associated hyperinflammation (7). Additionally, elevation in serum IL-18 has also been reported in patients with NOCARH syndrome (neonatal onset of cytopenia, autoinflammation, rash, and HLH) with mutations in the GTPase *CDC42* (8). Despite current treatments, the mortality rate of HLH is approximately 40%, thus, there is a dire need for more studies to delineate disease pathophysiology and provide novel therapeutics to improve survival.

This Research Topic, titled “*Towards a better understanding of HLH*”, includes one case report and 4 original research papers. These studies mainly focused on providing novel insights on the causes of primary HLH and secondary HLH (Brauer et al., Zeng et al.), development of computational models to predict the underlying cause and survival in patients with secondary HLH (Gao et al., Cheng et al.), and on the use of escalating doses of ruxolitinib in the treatment of patients with malignancy-associated HLH (Song et al.).

In an original research article in this Research Topic, Brauer et al. described several complex cases in a family of seven siblings from consanguineous parents that exhibited symptoms of metabolic syndrome, increased susceptibility to EBV infection, and EBV-driven development of lymphoma (9). Whole exome sequencing revealed homozygous variants in *RAB27A* (c.19G>T, p.(Asp7Tyr), *RAB27Am/m*), *FBP1* (Fructose-1,6-biphosphatase 1) and *ACAD9* (Acyl-CoA dehydrogenase family member 9). Interestingly, one patient displayed homozygosity for all these three variants, one was homozygous for *RAB27A* and *FBP1* but heterozygous for *ACAD9*, one displayed homozygosity for *RAB27A* and *ACAD9*, and the other siblings were either not analyzed for all three mutations or displayed heterozygosity for at least one variant. Further functional analyses performed by the authors demonstrated that this mutation in RAB27A causes around 50% reduced binding to MUNC13-4 and melanophilin (MPLPH) and leads to impaired NK and CD8 T cell cytotoxicity. The authors correlated this impairment in cytotoxicity to reduced control of Epstein-Barr virus (EBV) and predisposition to lymphoma (2/7) in this family. Cytotoxic T lymphocytes (CTL) from family members that were homozygous for *RAB27A* had impaired granule exocytosis. CTL heterozygous for *RAB27A* displayed slower to normal killing capacity, while cells with mutations in all three genes, *RAB27A, FBP1*, and *ACAD9* had an impaired killing capacity (one patient). Reconstitution of CTL with WT RAB27A from patients with homozygosity in *RAB27A*, *FBP1* and/or *ACAD9*, partially rescued exocytosis of lytic granules. More studies are warranted to further delineate the role of FBP1 and ACAD9 in cytotoxic immune cell function and to bridge between metabolic and immune cell disorders in the context of infections (e.g. EBV) and malignancy (e.g. lymphoma) in the presence of these mutations.

Due to its anti-viral activity, pegylated interferon (IFN)-a has been used for viral suppression in chronic hepatitis B (CHB) (9). In this Research Topic, Zeng et al. described a rare case of a 31-year-old male with CHB that developed systemic lupus ertythematosus (SLE) complicated with secondary HLH after being treated with pegylated IFNa-2b for almost three years prior to admission (10). According to the patient’s history, there was no indication of any autoimmune disorder prior to peg-IFNa-2b treatment. Upon admission, the patient suffered from headache, fever, and low white-blood cell counts. Symptoms worsened after treatment with ipuprofen and subcutaneous granulocyte-colony stimulating factor (G-CSF). Further tests revealed that the patient was positive for antinuclear antibody (ANA), anti-ds DNA, anti-nucleasome Ab, and anti-histone Ab, and direct Coombs’ test, thus, suggesting SLE. Furthermore, the patient developed hyperferritemia, cytopenias, elevated liver enzymes, high levels of soluble CD25, and bone-marrow biopsy revealed hemophagocytosis. The patient met diagnostic criteria of HLH-2004 and was treated with a high dose of methylprednisone combined with etoposide, cyclosporine, cyclophosphamide, and entecavir. Fever reversed to normal, and the patient was maintained on cyclophosphamide, reduced dose of cyclosporine, and prednisone. Almost two years later, the patient remained stable and was maintained on cyclosporine, entecavir, and calcium. Previous studies have described the development of SLE following treatment with pegylated interferon (10, 11). Additionally, secondary HLH have been reported in a patient treated with pegylated interferon (12). However, it is still unknown how pegylated interferon could induce SLE and HLH in this patient and the mechanism by which HLH is acquired. Further studies will be needed to determine the direct link between the development of HLH secondary to SLE following treatment with pegylated interferon.

Diagnosis of secondary HLH can be clinically challenging. In an attempt to better predict the factors in hematologic and rheumatic disease that are mostly related to secondary HLH, Gao et al. applied a multivariate logistic analysis method (11). In this study, analysis involved 175 patients (≥16 years old), median age of 43 (16-88), including 72 males (41.1%) and 103 females (58.9%). In this cohort, 52.9% developed sHLH secondary to hematologic diseases (e.g. lymphoma, leukemia, myelodysplastic syndrome, aplastic anemia), while 47.4% were secondary to rheumatic diseases (e.g. adult-onset Still’s disease (AOSD), SLE, dermatomyositis, rheumatoid arthritis, Sjogren’s syndrome, juvenile idiopathic arthritis). Interestingly, parameters predicting a correlation with rheumatic disease included younger females, higher platelet counts, higher ferritin, and EBV negative compared to with hematologic disease. Variables predicting hematologic disease as a cause for sHLH included higher soluble CD25, reduction in complete blood counts, EBV infection, and increased splenomegaly. In this prediction model, a score ≥ 4 suggested that HLH developed secondary to a rheumatic disease, while a score < 4 suggested HLH secondary to hematologic disease (sensitivity 85%, specificity 75%). As suggested by the authors, future analysis involving patients from multiple centers will be needed to further validate this model. Another study in this Research Topic by Cheng et al. aimed to use the ALBI (albumin-bilirubin) scoring system to predict liver dysfunction and survival in patients with non-Hodgkin lymphoma-associated secondary HLH (NHL-sHLH) with liver injuries (12). ALBI score was calculated as [(0.66 X log_10_ bilirubin (µmol/L)) – (0.085 X albumin (g/L))]. Further, ALBI grades were defined based on ALBI score as follows: ALBI grade I (score ≤ -2.60), ALBI grade II (-2.60 < score ≤-1.39), ALBI grade III (score > -1.39). The analysis included 168 patients with NHL-sHLH admitted in one center between 2014-2019. The authors demonstrated that combined ferritin levels to the ALBI score was a better predictor of 30-day to one year mortality than using either ferritin or ALBI score alone. Future studies combining a larger cohort of patients from multiple centers would be needed to further validate these prediction models.

Since many of the elevated cytokines in HLH signal through the JAK-STAT pathway, many preclinical and clinical studies demonstrated the efficacy of using JAK1/2 inhibitor, ruxolitinib, for the treatment of HLH (13–15). In this Research Topic, Song et al. applied a treatment regimen with dose escalating ruxolitinib for the treatment of 8 patients refractory for HLH (13). In these patients, HLH was secondary to autoimmune disorder, AOSD, SLE, myelodysplastic syndrome, pregnancy, or lymphoma. Four patients were identified as being heterozygous for *LYST* or *PRF1* mutations. In 4/8 patients, complete response (CR) was achieved after an escalated dose of ruxolitinib. Two patients only achieved remission after treatment with low-dose etoposide after an escalating dose of ruxolitinib. The remaining two patients died. Interestingly, poor responses to ruxolitinib were associated with malignancy and higher levels of sCD25 (>10,000 pg/mL). Patients with sCD25 higher than 10,000 pg/mL responded to an extra low dose etoposide. Therefore, the authors consider the use of additional chemotherapy in cases of malignancy associated HLH and in patients with sCD25 higher than 10,000 pg/mL. Furthermore, the authors did not report severe side effects when using a maximum dose of ruxolitinib of 30mg three times a day. Additionally, worsened cytopenias was not reported in patients treated with escalating doses of ruxolitinib. Larger cohorts of patients and multi-center trials will be needed to establish the use of ruxolitinib in the treatment of HLH.

Overall, the research articles in this Research Topic shed a light on novel causes of sHLH, such as HLH secondary to SLE following pegylated interferon treatment, new variants bridging impaired cytotoxicity and metabolic syndrome in siblings of consanguineous parents, novel prognostic models to predict whether sHLH would be associated with rheumatic or hematologic disease, the use of ferritin levels in combination with ALBI score to predict mortality in malignancy-associated HLH, and the use of escalating dose of ruxolitinib in patients with refractory sHLH.

## Author contributions

SA: Writing – review & editing, Writing – original draft.
